# Control under times of uncertainty: the relationship between hospital competition and physician-patient disputes

**DOI:** 10.1186/s12939-017-0701-0

**Published:** 2017-11-28

**Authors:** Qian Yang, Jay Pan

**Affiliations:** 1 School of Public Health, Zhejiang University School of Medicine, Hangzhou, China; 2 West China School of Public Health, Sichuan University, Chengdu, China; 3 West China Research Center for Rural Health Development, Sichuan University, Chengdu, 610041 China

**Keywords:** Medical dispute, Hospital competition, Social control, China

## Abstract

**Background:**

Recently, cases of medical disputes and even acts of violence toward physicians by patients in China have been escalating. It remains unknown whether competition improves the patient-physician relationship.

**Methods:**

This paper analyzes the relationship between hospital competition and the probability of medical disputes occurrence according to the theory of social control. Data from all hospitals in the Sichuan province of China from 2011 to 2014 were included in the study. The fixed radius approach with GIS information was employed to define hospital market, and the differences in competition over time and across regions were utilized. Our analysis is based on the fixed effect estimation, which accounts for the time-invariant unobserved heterogeneity among hospitals.

**Results:**

We found an inversed U-shaped relationship between HHI and the likelihood of medical disputes. As beneath either situation of monopoly or full competition, the burst of physician-patient dispute was downward into a valley, but it rises and then falls again with the increase of HHI, it reached the peak at the typical semi-market hospital competition structure.

**Conclusions:**

Our results highlight the probability of change in disputes occurrence with the transition of hospital competition and its psychological explanation, providing implications for China’s future health reform.

## Introduction

The ecology of Chinese medical practice has been characterized by a skyrocketing number of medical disputes and even outright violence toward physicians [1, 2]. In 2013, a total of 70,000 medical disputes were reported (National Health and Family Planning Commission).

The high occurrence of physician-patient disputes can reflect the current physician-patient relationship, echoing the state of chaos that exists in the healthcare market in China. An important goal for the new round of China’s health reform policies is to improve the efficiency of the delivery of healthcare throughout China, and shift the strategy to market competition. Chinese government has emphasized the role of market mechanisms in the new round of health reform. The private capital is encouraged to investing in the health care sector for the current health policy, driving the China’s health care market towards higher intensive of “competition” in the recent years [3]. However, the debates of whether competition does in fact improve health care market outcomes continue to be carried on [4–6].

Some have suggested that competition in the health care market effectively and broadly mobilizes production ([7–10]; Gaynor & Town, 2011; [5]). Also, it has been offered that competition should be more welcomed than antitrust, as captured by the statement made by Greaney [11]: *“Antitrust finds itself some twenty years later an unwelcome guest at the wedding as state and federal legislators prepare to embrace competition in their overhaul of the health care system”*. When hospitals wish to win out in cruel competition, they have to try their best to improve their quality of service in order to avoid both patients dissatisfaction and medical disputes. However, caution is urged as many have argued that hospital competition may result in a medical arms race, leading to higher prices for patients and unnecessary service utilization due to possible supplier-induced demands [12–14]. It also remains unknown whether competition will bring improvements to the patient-physician relationship.

Whether a monopoly or a competitive market is more advantageous is just a static comparison. But it might be more meaningful to abandon the static comparison and set it under a dynamic back group to analyze when medical disputes are pounding the Chinese health care market. With the process of reforms and open policy in the 1980s, the healthcare market experienced great transitions, which are dynamic processes that brought about major changes in regard to people’s livelihoods.

It is known that the semi-market state (market in transition) caused a lot of problems, such as lack of regulation in advertising, cost escalations, and lack of coordinated care for patients [15, 16]. A recent scandal in the Chinese health care market - the tragedy of Zexi Wei – reflected the negative impact brought by the increase in hospital competitios – which is related to the semi-market state [17]. With the increase of competition, hospitals have heavily invested in the information search rank of popular Chinese search engine Baidu. However, this 21-year old Chinese college student died following dubious treatment from a hospital’s advertisement on the Baidu site. His family borrowed money and spent over $30,000 for treatment, which only turned out to worsen his health status. The tragedy can be mainly attributed to the illicit competition among hospitals. This case is a true portrayal of the chaotic Chinese health care market that is characterized by a semi-market status, which is usually associated with inefficient governance and lack of control, which leads to defiant competition [18–21].

## Background

The rapid deterioration of the patient-physician relationship in China can be attributed to people feeling a lack of control due to being impacted by the macro social-economic environment. One of the most critical problems that exists in medical care can be explained as adaptations to the existence of uncertainty in the incidence of disease and the efficacy of treatment [22]. Humans always strive to perceive that they possess personal control over their social environments and outcomes, as this motivation helps to prevent feelings of randomness and chaos in the social world ([23]; Perkins Jr., 1968; [24]). Kay et al. [25] developed a model to explain the relationship between peoples’ compensatory control mechanism and their support for external systems of control.

There are debates regarding the psychological benefits between monopolistic and competitive markets. It is unknown whether a powerful monopolistic or a highly regulated full competitive market would be effective in providing feelings of control for a patient, thus compensating for feelings of chaos and uncertainty in the external system and finally decreasing the probability of medical disputes occurrence.

One side of the coin is the monopolistic market. The exclusive hospital has total market power individually to affect either the quality or other outcomes of health care in the market [26, 27]. The previously monopolized Chinese health care market can be perceived to possess the power of regulation and had high levels of certainty, as the government had high control over all of its activity. Thus, the powerful monopolistic health care market would become a source of personal control for patients.

The other side of the coin is the competitive market. A competitive health care market would establish a well-functioning system. Market players, including the producer and consumer, will trust in one another to decrease transaction costs and gain a reputation, as well as react to market order and rules. This is an opposite situation of chaos and randomness. It could conquer the feelings of uncertainty and lack of control of patients in the health care market.

However, a consensus has not been reached. In previous studies, we found that hospital competition would improve China’s health care delivery [5]. And in a more competitive market, there are fewer complaints and dissatisfaction from the patient side [28]. Nevertheless, some other studies argued that although the general rules of marketization in a competitive market claim that substantial demand increases will cause income distribution changes, resources would still be redeployed by the price mechanism [29]. The previous literature doesn’t do a very good job of interpreting the relationship between the competition of the health care market and the physician-patient dispute under China’s context of “*Kan Bing Gui, Kan Bing Nan* (getting medical care is expensive and difficult)” [30].

**Fig. 1:**
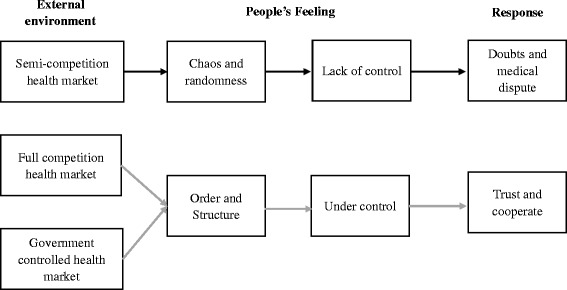
Conceptual diagram of the dual-response model of market and dispute, adapted from Kay et al. [25]

Because of the psychological need to insulate the self from feelings of randomness and chaos, people need a substitutability of the belief in personal control with the belief that things are under control. Meanwhile, just as a powerful government or system could suggest a reduction of randomness in the social order, it provides for people a way to compensate feelings of safety and a state of completion [25]; External systems with enough market power could suggest a reduction of medical disputes in the health care market as stated in Fig. 1.

The economic reforms in China have major impacts on political, cultural, and various other societal spheres, in a large part affecting people’s feelings of uncertainty. This can be seen in the physician-patient relationship, which is nowadays dependent poor legal and financial mechanisms, instead of on alternative financing channels and governance mechanisms, such as those based on reputation and relationships [18]. The nature of Chinese economic transactions has contributed to an economic environment full of uncertainty and disorder, causing the broader social system to also inevitably appear disordered. In this state, people are more likely to attribute the influence over life events to an enemy figure [31]. This effect has been demonstrated in the medical field, when during situations of ambiguous diagnosis, patients are more likely to place blame on and be angry toward physicians instead of finding fault in themselves or in fate [32].

Throughout the history of medicine there exists a consistent belief that crosses cultures that the medical practice has been perceived by people to be associated with religion, salvation, and related beliefs [33]. Justly so, the ancient patient-healer relationship in China is closely correlated with selflessness and universal love, as Sun Szu-miao (AD581–682), a famous physician, Taoist and alchemist, wrote in his monograph entitled *On the absolute sincerity of great physicians*. He wrote that healers should commit oneself with great compassion to save every living creature, and treat everyone on an equal basis, regardless of economic status [34].

Even during the earliest period of Chinese economic reform, this folk bioethic and values orientation were inherited in the socialism medical morality and cosmic. However, this belief was considered a “morality compulsion” by western viewers [35]. After China stepped into the industrial society, and was affected by the atmosphere of western culture, marketization, and modernization, the physician-patient relationship also shifted according to the epoch.

Together with the modern experience, traditional systems of stratification have been eroded, if not abolished, and traditional moral orders feel less binding than they did before. People are more mobile than before, and this has implications for the sense of connectedness that they feel with each other. At the same time though, situated in the midst of this flux, in a world that feels as though it is both falling apart and yet still hanging together, many people feel vulnerable and seek a sense of security and uncertainty [36]. The management of this deeply ingrained, existentially based “ontological insecurity” was considered a key component of late-modern citizenship [37].

We have to admit that, when we are faced with a society where traditions, bonds, and orders are becoming increasingly fragile and fragmented, no matter cognitively or affectively, people will start to rely more on social control agents that can provide social control. Especially in an eastern culture such as China, the theory of social control could be utilized to interpret the association between peoples’ lack of control and the frequently occurrence of medical disputes or even violence attacks against doctors. At this point, people tend to use the formal state apparatus of social control [38]. As such, the theory of social control could be utilized in our study to interpret the association between peoples’ feelings of lack of control and frequently occurring number of medical disputes and violent attacks toward doctors.

There are two kinds of control: *hard edge* and *soft edge* [39]. The *hard edge* is characterized by coerced controlling actions, and the *soft edge* of social control consists of more psychological and therapeutic forms of diagnosis, persuasion, and intervention without coercive measurements.

At the market level, the powerful existence for both the monopolistic and competitive market is regarded as compensation for peoples’ psychological lack of control. The beneficial effects of monopolistic and competitive health care market rely on *soft* control instead of a hard one. Only when the competitive hospital market is regulated enough or has enough control over all its agents, via means of soft control, does it have the ability to earn complete trust from people. On the other hand, when the market agency is in the monopoly position, compensation mechanisms of soft control can occur, also increasing people’s feeings of trust and reducing the possibility of violence. These two conflicting expectations, of whether more competition or less competition would decrease the medical disputes occurrence, presents an unknown. When the theoretical analysis cannot provide a definite answer, we need the empirical data to verify it. Fortunately, China is a proper experimental field of doing so since it is still in the process of economic transformation.

In the present study, we explore the relationship between hospital market competition, the level of which has varied over time and region, and the probability of medical disputes occurrence in Sichuan Province, by employing the fixed radius approach with GIS information to define the hospital market. This study goes beyond previous studies, which have generally reported case studies or macro-level data, by using hospital level data on medical disputes.

## Methods

### Data

Using several government sources, data from 2011 to 2014 in all hospitals in Sichuan Province, including public and private facilities, and at primary, secondary and tertiary levels, were included in our analysis.

Sichuan Province represents a typical “China’s province”, of which land area ranks fifth in China, with a population of more than 80 million [40]. First, like the whole country, the province’s distribution of economic development is uneven. Sichuan has both relatively more developed regions like Chengdu and Deyang, as well as poorer regions like Bazhong and Liangshan. Second, the geographic environment is varied, also according with the national situation. Eastern Sichuan mostly consists of a large plain. Mid-Sichuan is hilly, and Western Sichuan is mountainous. Third, its composition of the population is diverse, which is consistent with national demographics. Sichuan contains large populations of many different ethnic groups including the Qiang in Aba, the Tibetan in Ganzi, and the Yi in Liangshan. This richness of differences among regions creates an excellent natural experiment for a study on the variation of hospital competition within the province, a microcosm of China, which will help us better understand the relationship between hospital market competition and medical disputes of the whole society.

We included hospitals both in horizontal and vertical levels. In the horizontal level, we collected data from public and private hospitals, as well as general hospitals, traditional Chinese medicine (TCM) hospitals, and specialized hospitals. In the vertical level, we obtained information from primary, secondary, and tertiary hospitals.

In sum, the current study covered all types and levels of hospitals listed above from 181 counties in the Sichuan Province. Observations with missing data for key variables were excluded. This leads to a final study sample of 6262 hospital-year observations during 2011–2014, corresponding to 1857 hospitals. The longitudinal nature of the sample enables us to adopt the panel data econometrics to eliminate the impact of hospital heterogeneity and the associated estimation bias.

### Variables

The outcome variable in this study is the likelihood of medical dispute for a specific hospital. We treat it as a dummy variable, where the value equals one, if any, medical dispute that happened in the past year, and zero otherwise. According to the Health and Family Planning Commission of Sichuan Province, in our database, the medical dispute denotes the events in which a patient and/or patient’s relative(s) is dissatisfied with the care and treatment received by the hospital staff, thus they require the health administrative department or the judicial authority to investigate or compensate for the losses (including outpatient treatment and inpatient stay). Our hospital market competition measure is the Herfindahl–Hirschman Index (HHI) within a certain geographic area by year. HHI has frequently been used in industrial organization theory to represent the dispersion of firms within one industry, as well as being the most commonly used variable to measure degrees of competition in the empirical literature [8, 10].

At first, we employ the fixed radius approach to defining the hospital market by assigning a unique market area to each hospital, within which the region was enclosed by a circle centered on the hospital ([41]–1985).^1^ Following the literature [42], in our main regressions, we define the hospital’s catchment area as 15 miles. We got the distances between any of the two hospitals by GIS with information of the hospitals’ latitude and longitude.^2^

Therefore, HHI was constructed within the catchment area by the following equation:

1 $$ {HHI}_{it}=\sum \limits_{h=0}^N{\left({X}_{ht}/{X}_{mt}\right)}^2=\sum \limits_{h=1}^N{S_{hmt}}^2 $$

where *i* denotes the specific hospital, *m* is the hospital market defined by the fixed radius approach according to hospital *i*, *h* is the hospital within the hospital market *m*, *t* is time, *N* indicates the number of hospitals within the market *m*. *X*_*ht*_ is the total revenue of hospital *h* in market *m* in year *t*, *X*_*mt*_ is the total revenues of hospital market *m* in year *t*. *S*_*hmt*_ is the market share of hospital *h* in market *m* in year *t*. *HHI*_*it*_ measures the degree of competition faced by hospital *i* in a given year *t*. Smaller values of *HHI* indicate lower values of market concentration, thus higher levels of competition.

In addition to the key explanatory variable, a set of hospital-level characteristics and regional factors are included in our regression. Hospital-level characteristics included hospital ownership type (public vs. private), business propose (profit vs. nonprofit), service dimension (general hospital vs. specialty hospital), hospital rank (primary, secondary, or tertiary), hospital size (total number of hospital beds), and hospital scale (total number of visits per year). Three other variables are also included to capture the regional differences in the demographic, social and economic environment: total population, urban population ratio, and GDP per capita.

### Analytical strategy

In the results, we will first illustrate the bivariate relationship between the main independent and dependent variables.

Following the descriptive analysis, we will estimate the following regression model to test our main hypothesis.

2 $$ {Dispute}_{it}={X}_{it}^{\hbox{'}}{\beta}_1+{COMPETITION}_{it}{\beta}_0+{a}_i+{\varepsilon}_{it} $$

where, *Dispute*_*it*_ is the likelihood of medical dispute for hospital *i* in year *t*. *COMPETITION*_*it*_ is the proxy variable indicating competition degree of hospital *i* facing in the time period of *t*, the coefficient *β*_*0*_ is the average impact of the competition of interest. In addition, other factors which might influence outputs are also included in the model, as indicated by *X*_*it*_ to cover a series of control variables, including hospital characteristics, and regional economic, demographic, and social characteristics.

The model is quite straightforward, albeit with some estimation challenges. A major concern is the assumption of the composite error term (*a*_*i*_ + *ε*_*it*_). If *a*_*i*_ = 0, strict ordinary least squares (OLS) can consistently estimate the relationship between competition and medical dispute occurrence, *β*_*0*_. If *a*_*i*_ is itself a random variable, then for the composite error term, the random-effects model (RE) would be more efficient. The RE model takes on the form as below:

3 $$ {Dispute}_{it}-\lambda \overline{Dispute_i}=\left({X}_{it}^{\hbox{'}}-\lambda \overline{X_i^{\hbox{'}}}\right){\delta}_1+\left({COMPETITION}_{it}-\lambda \overline{COMPETITION_i}\right){\delta}_0+\left({\nu}_{it}-\lambda \overline{\nu_i}\right) $$

where, *υ*_*it*_ = *a*_*i*_ + *ε*_*it*_,, and $\lambda =1\hbox{-} {\left[{\delta}_{\varepsilon}^2/\left({\delta}_{\varepsilon}^2+\mathrm{T}{\delta}_a^2\right)\right]}^{1/2}$.

But if *a*_*i*_ is endogenous when unobserved heterogeneity among hospitals, such as hospital management practices, is associated with both competition and our outcome variables, the OLS and RE would be biased, leading to upward or downward bias in estimating the coefficient of hospital competition.

In this case, if the unobserved hospital effect factors do not change over time, we may get consistent estimation using the fixed effect model (FE) to control for this unobserved heterogeneity, under the assumption that the idiosyncratic error *ε*_*it*_ is strictly exogenous. The FE model takes on the following form:

4 $$ {Dispute}_{it}-\overline{Dispute_i}=\left({X}_{it}^{\hbox{'}}-\overline{X_i^{\hbox{'}}}\right){\theta}_1+\left({COMPETITION}_{it}-\overline{COMPETITION_i}\right){\theta}_0+\left({\varepsilon}_{it}-\overline{\varepsilon_i}\right) $$

By subtracting the time-series means of each variable for each hospital, the impact of any time-invariant fixed effects is eliminated, enabling the model to provide a consistent estimate of the relationship between competition and the likelihood of medical dispute occurrence.

This fixed-effects model must be interpreted with care, as it captures only the short-run effects of hospital competition variation that occur during the 4-year span of the data. Further, it may still yield biased estimation if the competition variations observed in our data themselves reflect feedback from medical dispute shocks. We believe, however, that such bias is unlikely that hospital-level conditions (hospital medical dispute occurrence) affected market-level conditions (hospital market competition).

Following the literature [43], to choose between an RE and FE, we further employ the Hausman test [44] to check the null hypothesis that *a*_*i*_ is exogenous.

## Results

### Sample description

Table 1 shows the summary statistics.^3^ The sample contains 6262 observations in 5138 hospitals, of which 2766 are public hospitals and 2417 private hospitals. The sample covered all three-level hospitals, with 68% primary hospitals, 26% secondary hospitals, and 6% tertiary hospitals. Table 1 presented the samples in different years. We could observe the Linear reduction trends both of medical disputes and HHI.

**Table 1:** Data description

Variable	No. (%)				
	Overall (*N* = 6262)	2011 (*n* = 1318)	2012 (*n* = 1502)	2013 (*n* = 1669)	2014 (*n* = 1773)
Any medical dispute happened in the hospital (Yes = 1)	2427	954	457	488	528
	(38.8)	(72.4)	(30.4)	(29.2)	(29.8)
HHI (15 miles radius), mean (SD)	0.280	0.291	0.284	0.277	0.271
	(0.205)	(0.218)	(0.213)	(0.201)	(0.192)
Public hospital (Yes = 1)	2766	685	694	691	696
	(44.2)	(52.0)	(46.2)	(41.4)	(39.3)
For-profit hospital (Yes = 1)	2414	434	544	683	753
	(38.5)	(32.9)	(36.2)	(40.9)	(42.5)
*Hospital rank*					
Primary (Yes = 1)	4252	866	1018	1157	1213
	(67.9)	(65.7)	(67.8)	(69.3)	(68.4)
Secondary (Yes = 1)	1636	383	401	416	436
	(26.1)	(29.1)	(26.7)	(24.9)	(24.6)
Tertiary (Yes = 1)	372	69	83	96	124
	(5.9)	(5.2)	(5.5)	(5.8)	(7.0)
Comprehensive hospital (Yes = 1)	4202	892	992	1120	1198
	(67.1)	(67.7)	(66.0)	(67.1)	(67.6)
Total number of hospital beds, mean (SD)	171.887	160.200	171.184	173.101	180.027
	(286.534)	(260.539)	(280.953)	(291.019)	(304.771)
Total number of visits (10,000), mean (SD)	8.467	8.366	8.544	8.312	8.621
	(21.546)	(19.677)	(20.940)	(21.614)	(23.265)
Total population (10,000), mean (SD)	67.444	66.142	68.028	67.670	67.705
	(32.416)	(32.210)	(33.575)	(31.845)	(32.103)
Urban population ratio (%), mean (SD)	43.277	43.056	42.950	43.426	43.577
	(28.815)	(29.299)	(29.150)	(28.466)	(28.513)
GDP per capita (Yuan), mean (SD)	38,726.090	32,736.638	37,248.656	40,061.853	43,172.685
	(20,875.279)	(16,350.733)	(19,598.209)	(21,554.902)	(22,985.638)

### Main results

**Fig. 2:**
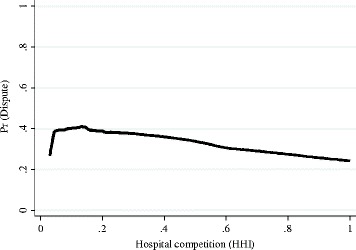
Lowess curve of the probability of dispute against hospital competition (HHI)

Figure 2 is the Lowess curve [45] of the mean probability of medical dispute occurrence against hospital competition. The curve illustrates a nonlinear relationship between competition and the mean probability of medical dispute occurrence, coinciding with our original hypothesis: at the left end of the curve first goes up quickly and then declines smoothly after reaching the peak, showing that the probability of dispute is relatively low when hospital competition is at its fullest or going toward monopoly. HHI indicates a state of full competition, where the dispute probability will be close to zero. Similarly, at the right end of the, the HHI indicates a state of monopoly, where the dispute probability will also be close to zero. The curve at the curve’s peak, where HHI indicates a relatively competitive market, dispute probability is also at its highest. Thus, we may describe the relationship between them as an inverted U-shaped relationship.

Since other confounders,such as hospital rank, type, and size may mix in the Lowess curves, the regression method was adopted to control for other unrelated variables better to understand the partial effect of hospital competition and medical dispute.

Table 2 presents the FE and RE estimation results. Based on the discussion in previous sections, hospital competition is likely to be endogenous in the regressions, and the RE estimates tend to be biased. We used the Hausman test to check the null hypothesis that the explanatory variables and the hospital-specific error term are uncorrelated. The results at the bottom of the table are rejections of the null hypothesis, and shows the differences in coefficients between the two models are systematic, that is endogenous, no matter the control variables are included. Thus, the estimation of the FE model is consistent. We focus on the FE results.

**Table 2:** regression results

Variables	Probability (Dispute)					
	(1)	(2)	(3)	(4)	(5)	(6)
	RE	FE	RE	FE	RE	FE
HHI (15 miles radius)	−0.009	1.675***	−0.101	1.503***	0.081	1.314***
	(0.121)	(0.348)	(0.096)	(0.346)	(0.128)	(0.346)
HHI square (15 miles radius)	0.115	−0.992***	0.073	−0.842***	−0.053	−0.701**
	(0.131)	(0.304)	(0.110)	(0.302)	(0.129)	(0.302)
Whether the hospital is public (Yes = 1)			0.117***		0.110***	
			(0.023)		(0.023)	
Whether the hospital is profit (Yes = 1)			−0.003		−0.001	
			(0.019)		(0.019)	
*Hospital rank*						
Secondary (Yes = 1)			0.340***	−0.071	0.297***	−0.046
			(0.030)	(0.058)	(0.031)	(0.057)
Tertiary (Yes = 1)			0.568***	−0.141*	0.381***	−0.061
			(0.030)	(0.073)	(0.056)	(0.075)
Whether the hospital is comprehensive hospital (Yes = 1)			0.021		0.013	
			(0.015)		(0.015)	
Total number of hospital beds					0.000***	−0.000**
					(0.000)	(0.000)
Total number of visits (10,000)					0.001	0.003**
					(0.001)	(0.001)
Total population (10,000)					−0.000	−0.004***
					(0.000)	(0.001)
Urban population ratio (%)					−0.000	−0.013***
					(0.000)	(0.002)
GDP per capita (Yuan)					0.000***	0.000*
					(0.000)	(0.000)
Year dummies	No	No	Yes	Yes	Yes	Yes
*N*	6262	6262	6262	6262	6262	6262
Hausman test results (*p*-value)	<0.001		<0.001		<0.001	

(1) Robust standard errors in parentheses; (2) ***p < 0.01, **p < 0.05, *p < 0.1; (3) RE denotes random-effect model, and FE fixed-effect model

The coefficients of both HHI and HHI squared variables were found to be statistically significant at 5% level, no matter controlling for all other potential confounding variables. The positive coefficient of the HHI and negative coefficient HHI squared indicate an inverted U-shaped relationship between HHI and the likelihood of medical dispute. When we calculate the HHI curve, we found that there is an inflection point 0.937 [−1.314/ (−0.701*2)], which represents the most serious physician-patient dispute.

This result implied the process of medical dispute variates according to the revolution of hospital market structure. From most monopoly (when HHI value as 1) to the breakup of monopoly, the likelihood of medical dispute is increasing. The probability of medical dispute reached the peak value as a HHI of 0.937. However, after that, with increasing marketization, the probability of medical dispute continues to decrease smoothly.

We did not find any evidence that the features of a hospital are associated with increased likelihood of medical dispute. Although there is a negative index for hospital rank, which indicates that a lower probability of medical dispute occurring in higher rank hospitals (e.g. secondary or tertiary hospitals), this was not statistically significant (*p* > .05). The difference between hospital scale and medical dispute can be captured by the number of beds. A large number of beds can further dilute the probability of dispute. In China, it can also be explained by the resource concentration in big hospitals. They possessed doctors with better medical skills, more advanced equipment, and a lower probability of medical dispute. However, if the hospital scale was controlled, the likelihood of medical dispute increased significantly with the total number of visits (*β* = 0.003, *SE* = 0.001, *p* < .05). When the admissions to the hospital increased 100,000 annually, the probability of medical dispute incremented by 3%.

The regional characteristics statistically affect the likelihood of dispute. The hospital has a lower probability of medical dispute if located in regions with denser population (*p* < 0.01), higher urbanization (*p* < 0.01), and less developed economy (*p* < 0.1).

### Robust test

Since variations of catchment areas definition for hospital markets would cause differences in measuring hospital competition, following Bloom et al. [8], we conducted robustness tests using alternative measures of hospital fixed radius, including 20 and 30 miles radius, respectively.

Table 3 shows how HHI and HHI squares are affected by the choice of the hospital market catchment areas. The 5% significantly positive and negative coefficients of HHI and HHI squares are found in all the FE models. Further, we find that the turning point varies from 0.762 for a 30-mile radius to 0.885 for a 20-mile radius. This narrow interval suggests that our results a relatively robust to the cut-off point which we used to define the catchment for hospital markets.

**Table 3:** Robust tests results

Variables	Probability (dispute)	
	(1)	(2)
HHI (20 miles radius)	1.328***	
	(0.378)	
HHI square (20 miles radius)	−0.750**	
	(0.339)	
HHI (30 miles radius)		1.481***
		(0.511)
HHI square (30 miles radius)		−0.972**
		(0.469)
Other control variables	Yes	Yes
*N*	6262	6262

(1) Robust standard errors in parentheses; (2) ***p < 0.01, **p < 0.05, *p < 0.1; (3) RE denotes random-effect model, and FE fixed-effect model

## Discussion

The results demonstrate that there is an interaction between hospital competition and the probability of medical disputes. The reversed U- shape relationship verified the model, which is reflected in Fig. 2. The probability of medical dispute reached the peak value as a HHI of 0.937 of the curve, makes it a not typical but still approximate U curve. Although the trend is limited in the real world, it still implied us that as beneath either situation of monopoly or full competition, the burst of physician-patient dispute was downward into a valley, but it rises and then falls again with the increase of HHI, it reached the peak at the typical semi-market hospital competition structure.

In the last decades, the health care market has changed dramatically concomitant with the economic transformation of China. Additionally, with the expeditious rise of per capita GDP, as observed in China, comes a range of potential issues including ecological problems [46], psychological problems [47] and social issues including increased levels of violence [48]. The relationship between economic transformation and conflicts that exists within the health care market in China is worth to study and mirror, despite claims from several literatures stating that China presents itself as a unique condition characterized by its political system and related developments. Nonetheless, reforms taking place in China can be taken for another country to consult during times of economic transformation, social development, and political changes.

While the levels of medical dispute in China are relatively high compared to other countries, they are by no means a problem unique to China and in many countries increasing rates are also observed. For example, the National Health Service Litigation Authority (NHS LA) of UK received 111 notifications of disputes across all health professions (including GPs, dentists, and opticians) in 2013/14; this is higher than the 80 disputes reported in 2012/13 [49]. Similarly, American Dietrich Healthcare reported that, in 2015, medical malpractice payouts increased over 4% from the previous year for the second consecutive year [50]. However, the most significant increase of medical disputes in other countries mainly appeared during the economic social transition, economic stagnation, or during the period of health care reform. For example, the steep slope of medical disputes in Japan happened in the late of 1960th [51], while the accelerate of American medical disputes started from 1970th [52].

Much of the literature around medical disputes, mostly from the 1990s, has focused on patients’ views and experiences [53–56]. More recent work on Chinese medical disputes has focussed on investigating the behavior of physicians or medical professionals (e.g. [1, 57]), attributed medical disputes as the result of a confluence of inappropriate incentives in the health system, the consequent distorted behaviors of physicians, mounting social distrust of the medical profession, and institutional failures of the legal framework. Trust crisis is the direct cause of disputes. However, the next question is what caused the trust crisis besides medium-level and micro-level. We believe that characteristics of the social transition period should take responsibility of the trust crisis. Further, the macro-economic structure is a direct expression of it. However, limited studies have researched the potential influence of changes in the macro social-economic environment, which will impact on the social mass’s psychological variation and finally increase the probability of extreme behaviors.

Despite the outright physician-patient disputes and even violence against doctors in recent years [2], three Chinese studies on physician-patient disputes summaries, the proportion of the incidence of physicians’ malpractice is below 10% [58–60]. It seems that the most likely reason behind the high rates of disputes is psychological changes caused by the macro social-economic transition, and not due to lagging behind on economic or other objective reasons such as medical technical advancement. Thus, this study adopts uncertainty theory of social psychology to investigate the relationship between hospital competition and medical disputes.

As mentioned by World Development Report [61] “*Competitive markets are the best way yet found for efficiently organizing the production of goods and services*”, our study supported this, by showing that the occurrence of medical disputes was higher with lower levels of health care market competition.

Second, it verified the power of monopoly as a sense of authority by providing social control in the face of potential uncertainty. Thus, when patients are suffering the uncertainty of the disease [62], and stayed in the health care market full of risk [63] in an era of uncertainty, the only redeemer they could think of is the monopoly institute.

Perceptions of chaos can arise during times of economic uncertainty [64, 65]. Monopolizing the healthcare market can decrease this perception of chaos by conpensating people’s lack of control with the control of the health care market, making people feel more safe and better off. This kind of external system of control presents a similar effect to other external systems of control like the government and religion, which has been shown to provide illusory feelings of control [25].

During China’s dramatic change of macro-social environment, there are many uncertain and demanding parts of the social change process [66]. The psychological consequences of the transition to a market economy in Chinese hospitals have been significant for both of physicians and patients. In the transition course of hospital market from monopoly to competition, both monopoly power and market rules are weak states and neither side can provide control over the patient-physician relationship. Since humans have a psychological need to perceive that they possess personal control over their social environments and outcomes, the management of this “ontological insecurity” brought by late-modern citizenship [37] must be achieved as well [23–25, 67–69].

This could also be verified in other nations. The UK was known as a single-payer health care [70], while the American medical system is famous for its marketization. Are there any correlations between the degree of market competition and feelings of perceived control? Generally speaking, the competitive market is antithetic to the monopolistic market [71]. It is a puzzle between monopolies versus market: one point of view supports the government intervention and the domination of public hospitals; the other supports market intervention, through private hospitals.

As a single-payer healthcare system, the National Health Service (NHS) in the UK is closely associated with the Keynesian welfare settlement of the 1940s [72]. While the health care system in the United States is in favor of a gentle “Third Hand” to assist the *Invisible Hand* of Adam Smith [73], the UK has a long history of intervention by central government, marked by reforms to make the system more competitive and cost effective [74, 75]. The reform of the quasi-market-based system allowed some hospitals to opt out of control by local administrative bodies and become independent self-governing trusts [76, 77]. For the United States, the enacted Patient Protection and Affordable Care Act of 2010 envisions a significant increase in federal oversight over the nation’s health care system. Although the legislation requires the states to play key roles in every aspect of the reform agenda, the nation’s fragmented and decentralized system are likely to continue.

The current research has advantages on measurement of the market competition and monopoly compared with empirical studies on Chinese medical market competitions. This is the first study to employ the fixed radius approach to define the hospital market in China. Instead of defining the hospital market according to the administrative divisions, as previous research did [5, 28, 78], the adoption of a fixed radius approach, defined medical market more accurately by GIS information reflects both the number of hospitals and the market shares of hospitals [42], which also keeps consistent with the international frontiers (e.g., [8]). Moreover, we take the HHI index to define the structure of health care market. It’s different to previous research which used the ratio of private hospitals to public hospitals in the market [78]. The latter only considered the competition between hospitals with opposite property rights, but ignored hospital competitions within a more wide-spread scope (such as the competition among public hospitals) [3]. As an index more frequently used in the empirical studies of aggregate hospital behavior and in antitrust cases, HHI reflects both the number of hospitals and the market shares of hospitals, and maps out the real reason for the high incidence of physician-patient disputes.

## Conclusion

China’s rapid economic growth has resulted in it becoming one of the most industrialized countries in a very short time span. The experience of industrialization and modernization provided us with a perception of a world of continuous change that is striding forward [79]. Chinese people have to deal with the pressure and challenges brought about by these changes. However, with dramatic developments in the economy, there is no compatible sudden shift in political structure and economic policy. The ultimate success of China will depend on its ability to create stable, efficient rules and regulations which create feelings of control and certainty for people to cope with social pressures, and to upgrade the equality and quality of health care services.

Many countries all over the world are in the midst of adjusting the structure of their health care markets. The role of government control and free market competition are two important ideologies. The key to health care market construction is adjusting measures to local conditions. Methods include giving enough authority to the government public welfare work, or giving the full play of market competition after setting up the market order. No matter which method is chosen, the possibility of medical disputes can be decreased by guaranteeing people’s feelings of control. However, a mixture or semi-market competition appeared to be linked with higher levels of medical disputes and should be avoided.

Understanding more about the reform of the economy and health system processes will allow us to interpret much about the role of governments, culture, and other agencies in the market transition from monopoly to competition - a transition characterized by an uncertain environment that impacts everyone in the health care market. The social problems brought by rapid economic development and social transformation are generally universal for any country or region, which makes the case of China a valuable example to be referenced.

Nevertheless, we recognize that this study has a number of limitations. First, the empirical strategy used in this study cannot fully resolve the endogeneity problem in hospital competition, particularly the endogeneity arising from the simultaneity between disputes likelihood and hospital market structure, as well as the time-variant unobserved factors, e.g., local governance, which affected both of the dispute and competition. Second, a dummy variable indicating the probability of medical dispute occurrence is used, and the number of dispute occurrences are not included in this study due to the limitation of administrative data availability. Considering our binary measurement of medical dispute could not reveal changes of patients’ right-protection awareness over time, we have added “Year” as a dummy variable in our regression to detect more truly results by controlling its effect. Third, the radius calculation is only applied to the unstable health market. Although it’s a popular method to measure regional competition in many references (e.g. [42]; and [8]), it’s not recommended to indicate competition after the hierarchical diagnosis treatment system would have been established and functioned. Since at that time, different hospital owns different functions, they could complement each other instead of competing. In future research, more comprehensive data may provide a more robust measurement and help identify the casual relationship.

## Footnotes

1 Fixed radius approach is a proper index to define the hospital market. It not only had the strength of healthcare utilization feature consideration, but also overcome the drawbacks of definitions based on geopolitical boundaries. Under the fixed radius approach, every hospital was assigned a unique market area, which is the region enclosed by a circle centered on the hospital and defined by a fixed radius. The fixed radius approach has the advantage of including a hospital’s nearby competitors, even when they are located on the other side of a political boundary.

2 The latitude and longitude of hospital locations were geocoded using the hospital names and addresses [40].

3 According to our statistical analysis, no matter the hospital is designated as the hospital for public health insurance or not, the regression results is always solid. If readers are interested in our data, we could provide it upon request.
